# Parameter Analysis of Multiscale Two-Dimensional Fuzzy and Dispersion Entropy Measures Using Machine Learning Classification

**DOI:** 10.3390/e23101303

**Published:** 2021-10-03

**Authors:** Ryan Furlong, Mirvana Hilal, Vincent O’Brien, Anne Humeau-Heurtier

**Affiliations:** 1 Institute of Technology Carlow, Carlow, Ireland; ryan.furlong@itcarlow.ie (R.F.); vincent.obrien@itcarlow.ie (V.O.); 2 LARIS—Laboratoire Angevin de Recherche en Ingénierie des Systèmes, University of Angers, 49035 Angers, France; mirvana.hilal@univ-angers.fr

**Keywords:** biomedical data, classifier, complexity, dispersion entropy, fuzzy entropy, entropy, irregularity, image analysis, multiscale approach

## Abstract

Two-dimensional fuzzy entropy, dispersion entropy, and their multiscale extensions (${\mathrm{MFuzzyEn}}_{2D}$ and ${\mathrm{MDispEn}}_{2D}$, respectively) have shown promising results for image classifications. However, these results rely on the selection of key parameters that may largely influence the entropy values obtained. Yet, the optimal choice for these parameters has not been studied thoroughly. We propose a study on the impact of these parameters in image classification. For this purpose, the entropy-based algorithms are applied to a variety of images from different datasets, each containing multiple image classes. Several parameter combinations are used to obtain the entropy values. These entropy values are then applied to a range of machine learning classifiers and the algorithm parameters are analyzed based on the classification results. By using specific parameters, we show that both ${\mathrm{MFuzzyEn}}_{2D}$ and ${\mathrm{MDispEn}}_{2D}$ approach state-of-the-art in terms of image classification for multiple image types. They lead to an average maximum accuracy of more than 95% for all the datasets tested. Moreover, ${\mathrm{MFuzzyEn}}_{2D}$ results in a better classification performance than that extracted by ${\mathrm{MDispEn}}_{2D}$ as a majority. Furthermore, the choice of classifier does not have a significant impact on the classification of the extracted features by both entropy algorithms. The results open new perspectives for these entropy-based measures in textural analysis.

## 1. Introduction

Information theory, relative entropy, and the Kullback–Leibler divergence are now widely used concepts (see, e.g., References [1,2,3]). Entropy-based algorithms have enabled engineers and researchers to measure the uncertainty and irregularity of complex systems and data [4,5,6]. The corresponding algorithms have become a key tool in many application areas, particularly in the biomedical domain. Thus, one dimensional entropy measures, e.g., sample entropy (SampEn${}_{1D}$) [7], permutation entropy (PerEn${}_{1D}$) [8], fuzzy entropy (FuzzyEn${}_{1D}$) [9], and dispersion entropy (DispEn${}_{1D}$) [10], have been proven effective at quantifying the irregularity of time series data. This success has led to the development of bidimensional (2D) entropy measures for images (2D data): SampEn${}_{2D}$ [11], PermEn${}_{2D}$ [12,13], FuzzyEn${}_{2D}$ [14], and DispEn${}_{2D}$ [15]. By being able to estimate the predictability or uncertainty of spatial patterns within images, entropy methods can be considered as effective feature extraction techniques. In recognizing that the repeatability of pixel patterns is related to the texture properties of images, entropy techniques can be employed in the analysis of textures within images, which in turn can be used to classify images. Classification from texture analysis has important applications in a large variety of fields such as medical image analysis, remote sensing, content-based image retrieval, object recognition, and many others (see, e.g., [16,17,18,19,20]).

The introduction of multiscale approaches to entropy measures arose from the need to quantify complexity in systems and to overcome certain limitations of single-scale approaches [21]. In the 2D multiscale approach, a coarse-graining procedure is applied to an image over spatial scales and then the entropy value is calculated for each coarse-grained version of the original image. The multiscale approach allows us to quantify the complexity of an image, where the complexity is defined as a measure of irregularity over several spatial scales.

Both FuzzyEn${}_{2D}$ and DispEn${}_{2D}$ (and their multiscale versions: MFuzzyEn${}_{2D}$ and MDispEn${}_{2D}$) use a set of tuneable parameters. In the case of fuzzy entropy, the parameters are *m* (the template length or embedding dimension), *r* (the matching threshold), and *n* (the fuzzy power). In the case of dispersion entropy, the parameters are *m* (the embedding dimension) and *c* (the number of classes). Understanding the sensitivity and impact of these parameters is an essential part in deploying these measures in biomedical applications.

An analysis of prior work in the area highlighted the fact that the majority of papers using MFuzzyEn${}_{2D}$ or MDispEn${}_{2D}$ employed parameter values based on tests performed on synthetic data. Additionally, no specific work has been carried out on the influence of these parameter values on classification results; see, e.g., Reference [22]. The goal of our paper is therefore two-fold: (i) to study the impact parameters have on FuzzyEn${}_{2D}$ and DispEn${}_{2D}$ values obtained from different image types and, by this, on image classification results; and (ii) to discern how different image types may lead to the selection of different sets of parameters to attain the most accurate classification results. For this purpose, we used two publicly available datasets: the Epistroma dataset [23] and the KTH-TIPS dataset [24]. To carry out the task of image classification, the entropy algorithms were combined with a set of commonly used machine learning classifiers. The use of multiple classifiers is significant to the study as it allows us to determine the impact of the classifier on the classification accuracy. From our review of the literature, we established that this work is the first to study the impact of combinations of parameter values on several datasets. It is also the first study on the influence of the parameter values with a classification approach.

The remainder of this paper is organized as follows: Section 2 presents the datasets processed as well as the algorithms and classifiers used. The results of the study are detailed and discussed in Section 3. Finally, the conclusion section at the end of the paper provides a summary of the results obtained.

## 2. Materials and Methods

### 2.1. Datasets

The Epistroma dataset [23,25] was employed. This dataset consists of histological images of colorectal cancer from 643 patients enrolled at the Helsinki University Central Hospital, Helsinki, Finland, from 1989 to 1998. The tissue samples have been stained with diaminobenzidine and hematoxylin, and were labeled into two classes: epithelium (825 samples) and stroma (551 samples). The dimension of the images varies from 172 × 172 px to 2372 × 2372 px. Our goal in this study was to analyze the classification capabilities and optimum parameter selection for ${\mathrm{FuzzyEn}}_{2D}$ and ${\mathrm{DispEn}}_{2D}$ (and their multiscale versions) in extracting texture features from biomedical images. In addition, we wanted to identify epithelium from stroma tissues using the proposed measures. For more information about the dataset, please refer to [25].

The other employed dataset was the KTH-TIPS dataset [24,26]. This dataset is composed of images of ten different materials (aluminum foil, bread, corduroy, cotton, cracker, linen, orange peel, sandpaper, sponge, and styrofoam). There are 81 images per material class, consisting of 9 different scales, 3 illumination differences, and 3 poses. The dimension of each image is 200 × 200 px. The target was to assess the classification capabilities and optimum parameter selection for ${\mathrm{FuzzyEn}}_{2D}$ and ${\mathrm{DispEn}}_{2D}$ (and their multiscale versions) in extracting generic texture features. For more information about the dataset, please refer to [26].

### 2.2. Experimental Procedure

#### 2.2.1. Data Pre-Processing

Images in the original datasets exhibit variations in their dimensions and color profiles. Therefore, prior to the feature extraction step, the following pre-processing steps were performed for all the images. Initially, images were imported into MATLAB R2020a [27]. To remove the variation in the image dimension, we calculated the center point of each image and cropped each image to two different sizes; these sizes were set at 50 × 50 px and 100 × 100 px, respectively. The second step involved taking the cropped images and transforming them into a grayscale representation, such that they had comparable ranges of pixel values. The grayscale transformation was performed instead of a standardization step as the grayscale transformations retain the texture information which is lost if the images are standardized in the case of our processed datasets. In addition to eliminating the variation, these steps reduce the computation time required by the entropy algorithms. After the pre-processing steps were complete, the images were divided into 75% training images and 25% test images.

#### 2.2.2. Feature Extraction

In our experiments, the entropy algorithms performed the feature extraction and dimensionality reduction step. The entropy algorithms were initialized with a set of parameters and the pre-processed images were then applied to them. For the single-scale approach, both ${\mathrm{FuzzyEn}}_{2D}$ and ${\mathrm{DispEn}}_{2D}$ algorithms output a single value representing the image. In the case of the multiscale approach, each algorithm outputs a vector of values representing the image. The resulting values of the entropy algorithms were then passed to the five classifiers and the classification accuracy was determined. The parameters used in the entropy algorithms were changed and the process was repeated. Each entropy algorithm was thus initialized with a combination of different parameters to examine the influence that different parameters could have on the quality of the extracted features and their classification performance.

#### 2.2.3. Fuzzy Entropy

FuzzyEn${}_{2D}$ has recently been proposed as an extension to the 1D fuzzy entropy algorithm [14]. By definition, FuzzyEn${}_{2D}$ is calculated as the negative natural logarithm of the conditional probability that two patterns similar for their corresponding m×m points will remain similar when the (m+1)×(m+1) points are considered. For FuzzyEn${}_{2D}$ calculation, initial parameters should be defined as follows: *m* as the template size, *r* as the tolerance level, and *n* as the fuzzy power.

Consider an image **U** = {u(i,j)} ${}_{i=1,2,.......,H}^{j=1,2,.......,W}$ of *H* × *W* size. At first, **X**${}_{i,j}^{m}$ is defined as the *m*-length square pattern of origin u(i,j), as follows:

(1) $$X_{i,j}^{m}=\left[\begin{array}{cccc} u_{i,j} & \ldots & u_{i,j+m-1}\\ u_{i+1,j} & \ldots & u_{i+1,j+m-1}\\ \ldots & \ldots & \ldots & \\ u_{i+m-1,j} & \ldots & u_{i+m-1,j+m-1} \end{array}\right].$$

Similarly, **X**${}_{i,j}^{m+1}$ is defined as the (m+1) square patterns. Let $N_{m}=\left(W-m\right)\left(H-m\right)$ be the total number of square windows in **U** that can be generated for both the **m** = [*m*, *m*] and **(m + 1)** = [m+1, m+1] sizes. For **X**${}_{i,j}^{m}$ and its neighboring windows **X**${}_{a,b}^{m}$, the distance function $d_{ij,ab}^{m}$ between them is defined as the maximum absolute difference of their corresponding scalar components. Knowing that *a* changes from 1 to H−m and that *b* changes from 1 to W−m with (a,b)≠(i,j), the distance function is expressed as follows:

(2) $$d_{ij,ab}^{m}=d\left[X_{i,j}^{m},X_{a,b}^{m}\right]=\max_{k,l\in (0,m-1)}(|u\left(i+k,j+l\right)-u\left(a+k,b+l\right)\left|\right).$$

The similarity degree $D_{ij,ab}^{m}$ of $X_{i,j}^{m}$ with its neighboring patterns $X_{a,b}^{m}$ is defined by a fuzzy function $\mu (d_{ij,ab}^{m},n,r)$:

(3) $$D_{ij,ab}^{m}\left(n,r\right)=\mu \left(d_{ij,ab}^{m},n,r\right)=\exp\left(-{\left(d_{ij,ab}^{m}\right)}^{n}/r\right).$$

Then, the similarity degree of each pattern is averaged to obtain:

(4) $$\Phi_{i,j}^{m}\left(n,r\right)=\frac{1}{N_{m}-1}\sum_{a=1,b=1}^{a=H-m,b=W-m}D_{ij,ab}^{m},$$

with (a,b)≠(i,j) to construct:

(5) $$\Phi^{m}\left(n,r\right)=\frac{1}{N_{m}}\sum_{i=1,j=1}^{i=H-m,j=W-m}\Phi_{i,j}^{m}\left(n,r\right).$$

It is similar for m+1 to obtain $\Phi^{m+1}\left(n,r\right)$. Finally, the bidimensional fuzzy entropy of the image U is:

(6) $$FuzEn_{2D}\left(U,m,n,r\right)=\ln\frac{\Phi^{m}\left(n,r\right)}{\Phi^{m+1}\left(n,r\right)}.$$

A key aspect of fuzzy entropy is that it employs, for the similarity degree, a continuous function (in our case $\exp\left(-{\left(d_{ij,ab}^{m}\right)}^{n}/r\right)$, where $d_{ij,ab}^{m}$ is the distance function [14]). The parameter *n* determines the gradient of the boundary of the exponential function and *r* is the width of the boundary of the exponential function, rather than the strict binary Heaviside function that is used by sample entropy measures.

Images with repeating periodic structures (regular patterns) would hold a low entropy value. On the contrary, images with non-repeating structures (irregular, unpredictable patterns) would hold a high entropy value. In what follows, we will use the notation of *m* as a scalar value for simplicity reasons. As we will choose squared embedding dimensions, the notation m=1 will represent [1,1], m=2 will represent [2,2], and so on. This notation will also hold for DispEn${}_{2D}$.

For FuzzyEn${}_{2D}$, we study the sensitivity of the following parameters on a range of values based on previous studies [9,14,28,29]: embedding dimension m={1,2}, tolerance r={0.12,0.24,0.36,0.48}, and fuzzy power n={2,3,4,5}.

#### 2.2.4. Dispersion Entropy

DispEn${}_{2D}$ [15] is our second employed entropy measure. In the latter, two initial parameters should be defined: *m*, the embedding dimension vector, and *c*, the number of classes. In DispEn${}_{2D}$, the values of the pixels within the image are mapped to *c* classes. This mapping results in embeddings (using an embedding dimension *m*) that are then matched to a dispersion pattern. When all possible two-dimensional dispersion patterns of an image have equal probability value, the highest value of DispEn${}_{2D}$ is reached, indicating irregularity in an image. The DispEn${}_{2D}$ algorithm is defined as follows. Consider an image **U** = {u(i,j)}${}_{i=1,2,.......,H}^{j=1,2,.......,W}$ of *H*×*W* size. First, u(i,j) elements are mapped into *c* classes using linear and non-linear methods [30] to form $z_{i,j}^{c}=round\left(c\times y_{i,j}+0.5\right)$. The number of classes *c* could be an integer from 3 to 9. In order to avoid having most of the u(i,j) elements be within the classes 1 to *c*, a sigmoid function is often used, where:

(7) $$y_{i,j}=\frac{1}{\sigma \sqrt{2\pi }}\int_{-\infty }^{u_{i,j}}e^{\frac{-{\left(t-\mu \right)}^{2}}{2\sigma^{2}}}dt,$$

with μ and σ representing the mean and standard deviation of the original image **U**. Let m be the embedding dimension vector [m,m] to define $z_{k,l}^{m,c}$ such as:

(8) $$z_{k,l}^{m,c}=\left[\begin{array}{cccc} z_{k,l}^{c} & z_{k,l+1}^{c} & \cdots & z_{k,l+(m-1)}^{c}\\ z_{k+1,l}^{c} & z_{k+1,l+1}^{c} & \cdots & z_{k+1,l+(m-1)}^{c}\\ \cdots & \cdots & \cdots & \cdots \\ z_{k+(m-1),l}^{c} & z_{k+(m-1),l+1}^{c} & \cdots & z_{k+(m-1),l+(m-1)}^{c} \end{array}\right],$$

where *k* ranges from 1 to h−(m−1) and *l* ranges from 1 to w−(m−1). After that, $z_{k,l}^{m,c}$ is mapped to a dispersion pattern $\pi_{\upsilon_{0},\upsilon_{1}\ldots \upsilon_{m\times m-1}}$. For each $z_{k,l}^{m,c}$, $c^{m\times m}$ dispersion patterns can be formed. Furthermore, the relative frequency is calculated for each of the $c^{m\times m}$ dispersion patterns, specifically $\pi_{\upsilon_{0},\upsilon_{1}\ldots \upsilon_{m\times m-1}}$:

(9) $$p\left(\pi_{\upsilon_{0},\upsilon_{1}\ldots \upsilon_{m\times m-1}}\right)=\frac{\#\{k,l,z_{k,l}^{m,c}\;\mathrm{has}\;\mathrm{type}\;\pi_{\upsilon_{0},\upsilon_{1}\ldots \upsilon_{m\times m-1}}\}}{\left(h-(m-1))(w-(m-1)\right)},$$

where l≤w−(m−1) and k≤h−(m−1). Finally, $DispEn_{2D}$ is calculated as:

(10) $$DispEn_{2D}\left(U,m,c\right)=-\sum_{\pi =1}^{c^{m\times m}}p\left(\pi_{\upsilon_{0},\upsilon_{1}\ldots \upsilon_{m\times m-1}}\right)\times \ln\left(p\left(\pi_{\upsilon_{0},\upsilon_{1}\ldots \upsilon_{m\times m-1}}\right)\right).$$

When the image is completely regular, the smallest value of DispEn${}_{2D}$ is obtained. In DispEn${}_{2D}$, two parameters must be manually selected: *m* and *c*. Combinations of m={2,3} and c={3,4,5,6} are all examined during our experiments. These values were chosen based on recommendations from Reference [15].

#### 2.2.5. Multiscale Approach

${\mathrm{FuzzyEn}}_{2D}$ and ${\mathrm{DispEn}}_{2D}$ allow to quantify the irregularity of images at one scale. However, such approaches are highly sensitive to high frequency components and may fail to account for inherent data at multiple scales [21]. To this end, multiscale entropy-based techniques have been introduced. These techniques can quantify the irregularity of an image over multiple spatial scales, defining its complexity [31,32]. These complexity-based measures are composed of two main steps: (1) a coarse-graining process, which involves removing high-frequency image components using a digital low pass filter and downsampling the filtered data by a scale factor τ; and (2) the calculation of an entropy method for each coarse-grained data at each scale τ. The multiscale extensions to ${\mathrm{FuzzyEn}}_{2D}$ and ${\mathrm{DispEn}}_{2D}$, known as multiscale ${\mathrm{FuzzyEn}}_{2D}$ (${\mathrm{MFuzzyEn}}_{2D}$) and multiscale ${\mathrm{DispEn}}_{2D}$ (${\mathrm{MDispEn}}_{2D}$), respectively, are used in this study. Thus, ${\mathrm{MFuzzyEn}}_{2D}$ and ${\mathrm{MDispEn}}_{2D}$ can be defined by the following two-step procedure:

1. Construct the coarse-grained images $I^{\left(\tau \right)}$ as
  (11) Iij(τ)=1τ2∑k=(i−1)τ+1l=(j−1)τ+1k=iτl=jτIkl,
  where $1\leq i\leq [\frac{H}{\tau }]$ and $1\leq j\leq [\frac{W}{\tau }]$.
2. Compute ${\mathrm{FuzzyEn}}_{2D}$ or ${\mathrm{DispEn}}_{2D}$ of each coarse-grained image.

A decrease in entropy values across spatial scales indicates that an image may be irregular but not structurally complex. However, when no noticeable changes in entropy values are observed across scales, this signifies that an image maintains complex structures across multiple scale factors; the image is said to be complex.

In our work, in the multiscale extensions for both entropy techniques, values of τ=1 to 10 were examined. This was performed to determine whether complexity analysis was better than irregularity quantification—that is, single-scale calculations—for improved classification outcomes.

#### 2.2.6. Classification

The extracted features were exported to Python, where the data was split into its respective 75% training and 25% test sets, in which classifiers from the Scikit learns library [33] were used to classify the texture features. The 5 classifiers used in this study are briefly outlined below.

##### Naive Bayes

The Naive Bayes classification methods are a set of supervised learning algorithms based on applying Bayes’ theorem with the “naive” assumption of conditional independence between every pair of features given the value of the class variable [34]. Bayes’ theorem states that given a class variable *y* and dependent feature vector $x_{1}\to x_{n}$ are guided by the following operation:

(12) $$P\left(y|x_{1},\cdots ,x_{n}\right)=\frac{P\left(y\right)P\left(x_{1},\cdots ,x_{n}|y\right)}{P(x_{1},\cdots ,x_{n})}.$$

In the Scikit learns *GaussianNB* algorithm, the likelihood between features is assumed to be Gaussian:

(13) $$P\left(x_{i}|y\right)=\frac{1}{\sqrt{2\pi \sigma_{y}^{2}}}exp\left(-\frac{{\left(x_{i}-\mu_{y}\right)}^{2}}{2\sigma_{y}^{2}}\right),$$

where the parameters $\mu_{y}$ and $\sigma_{y}$ are estimated using the maximum likelihood.

This variant of the Naive Bayes algorithm was employed with the parameters priors=None and var_smoothing=1e−9.

##### Decision Tree

Decision trees are a non-parametric supervised learning method used for classification and regression. The goal is to create a model that predicts the value of a target variable by learning simple decision rules inferred from the data features [35]. A tree can be seen as a piecewise constant approximation. The Scikit learns *DecisionTreeClassifier* algorithm was used in our experiments with the parameter random_state=0 and all other parameters were initialized with the algorithm’s default values.

##### Support Vector Machine

The main objective of the support vector machine (SVM) classifier is to find a hyperplane in an *N*-dimensional space, (where *N* represents the number of features) which bests discriminates classes [36]. The hyperplane is a decision boundary between data points, where the best hyperplane has the maximum distance between points in disparate classes. The equation for this decision boundary (hyperplane) is presented as:

(14) $$w^{T}x+b=0,$$

where *w* is the adjustable weight vector and *b* is the bias of the hyperplane. The linearly separable classes can be represented as follows:

(15) $$w^{T}x+b\leq 0\;for\;d_{i}=-1,\;w^{T}x+b>0\;for\;d_{i}=+1.$$

The Scikit learns *svm* algorithm was initialized with default parameters and was used in our experiments.

##### Multi-Layer Perceptron

Multi-layer perceptron (MLP) is a brand of artificial neural networks (ANN). MLP consists of three layers of neurons; an input layer, a hidden layer, and an output layer. Each neuron, except those found in the input layer, uses non-linear activation functions that transform the input of each neuron into a desirable output [37]. These networks are trained using back-propagation. The Scikit learns *MLPClassifier* algorithm was initialized with the parameters random_state=1 and max_iter=10,000, and all other parameters remained unchanged with their default values.

##### K-Nearest Neighbour

The K-nearest neighbour (KNN) is a type of instance-based learning or non-generalizing learning: it does not attempt to construct a general internal model but simply stores instances of the training data. Classification is computed from a simple majority vote of the k-nearest neighbors of each point: a query point is assigned the data class which has the most representatives within the nearest neighbors of the point [38]. The Scikit learns *KNeighborsClassifier* with values of k=1→9 was used in our experiments.

#### 2.2.7. Experimental Procedure

**Figure 1:**
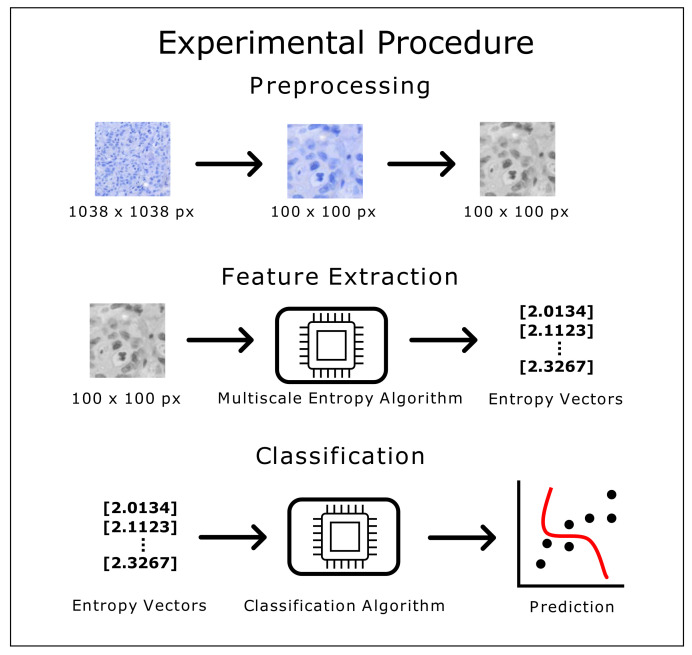
Flowchart depicting the different stages of the experimental procedure.

The experimental procedure was conducted using the following method: after pre-processing each image (reducing its size and converting it to grayscale), the feature extraction step involved applying the multiscale entropy algorithm (${\mathrm{MFuzzyEn}}_{2D}$ and ${\mathrm{MDispEn}}_{2D}$, independently) for each set of parameters. This led to obtaining the entropy values that were one set of values for each set of parameters. The number of entropy values calculated was dependent on the scale factor τ value. Thus, for instance, when τ=5, each image would be represented as a vector containing 5 entropy values. Then, these vectors would be independently exported to each classifier to obtain the classification results. The experimental flowchart used in our study can been seen in Figure 1.

## 3. Results and Discussion

In this work, the Epistroma dataset [23] and the KTH-TIPS dataset [24] were processed with two different multiscale two-dimensional entropy algorithms. For each algorithm, a different parameter combination was used. The corresponding entropy values obtained were used for machine learning classification purposes. The training/test data was split as 75% training and 25% test data. In all tests involving the Epistroma dataset, 1032 training and 344 test images were used, while 61 training and 21 test images were used for each unique texture in all the experiments involving the KTH-TIPS dataset.

### 3.1. Epistroma

The Epistroma dataset contains two classes representing two types of colorectal cancer tissue: stroma and epithelium. As such, a binary classification was used in the experiments conducted on the Epistroma dataset. Two different image sizes, namely 50 × 50 px and 100 × 100 px, were examined for each sample in the following experiments.

#### 3.1.1. Classification Accuracy

Initially, the multiscale entropy algorithms were compared based on their overall classification performance. The two classification metrics are average accuracy and average max accuracy. Average accuracy is the average of all classification results across all classifiers. Average max accuracy specifies the average of the highest classification accuracy achieved by each classifier. For this, we took the highest accuracy achieved by each of the five classifiers and averaged these values to derive a single result. Secondly, the optimal choice of each parameter was assessed by recording which parameter value resulted in the highest classification accuracy for each classifier. Finally, multiscale analysis was conducted to determine whether complexity or irregularity provides a better textural representation of our images. This was investigated by observing the τ value choice that results in the highest average classification accuracy for each classifier. If a τ value greater than 1 provides the highest average classification accuracy, this indicates that complexity provides a better representation of the texture in the images. Conversely, if a τ value equal to 1 provides the highest average classification accuracy, this indicates that irregularity provides a better representation.

**Figure 2:**
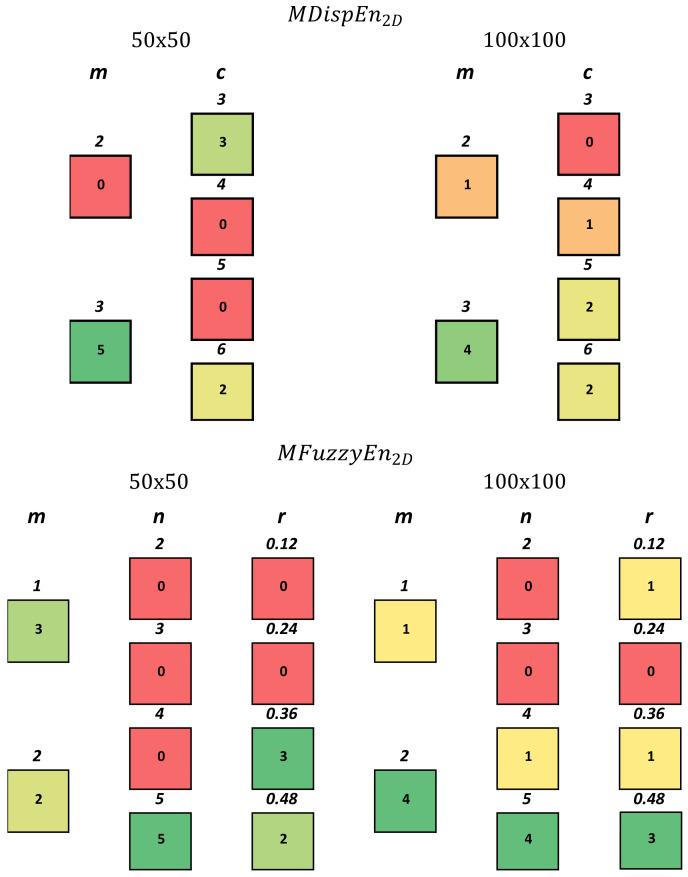
Color map displaying the performance of different parameter values as a representation of the classifier performance for the Epistroma dataset. The number located inside each box represents the number of classifiers (over five classifiers) that produced the highest average classification accuracy based on the respective parameter value (number above each box). Darker green boxes show the optimal parameter choice, while red boxes show the weakest choice.

1. *Classification Performance*: As shown in Table 1, across all possible parameter and multiscale combinations, ${\mathrm{MFuzzyEn}}_{2D}$ outperformed ${\mathrm{MDispEn}}_{2D}$ regarding average max classification accuracy for both image sizes, achieving accuracies of 98.84% and 100% for 50 × 50 px and 100 × 100 px images, respectively. However, ${\mathrm{MDispEn}}_{2D}$ achieved a higher average classification accuracy than ${\mathrm{MFuzzyEn}}_{2D}$ for both image sizes, producing accuracies of 93.66% and 94.77% for 50 × 50 px and 100 × 100 px images, respectively.
2. *Parameter Optimization*: Figure 2 shows a color map which displays the performance of different parameters with respect to each classifier for the ${\mathrm{MFuzzyEn}}_{2D}$ and ${\mathrm{MDispEn}}_{2D}$ algorithms. In the ${\mathrm{MFuzzyEn}}_{2D}$ case, for images of size 50 × 50 px, the value of *m* did not appear to have a significant influence on the classification performance, while a value of n=5 and larger values of *r* are recommended. Finally, for images of size 100 × 100 px, larger values for *m*, *n*, and *r* are recommended. In the ${\mathrm{MDispEn}}_{2D}$ case, for images of size 50 × 50 px, m=3 is recommended as all five classifiers performed optimally with this parameter value, while a *c* value of 3 or 6 is advised. Additionally, for images of size 100 × 100 px, larger values of *m* and *c* are favored.
  Additionally, in ${\mathrm{MFuzzyEn}}_{2D}$, parameter combinations of m=1, n=4, r=0.24,0.36,0.48, m=1, n=5, r=0.12,0.24,0.36 and m=2, n=5, r=0.36,0.48 resulted in an average accuracy greater than 85% for all the classifiers and both image sizes. For images of size 50 × 50 px using ${\mathrm{MDispEn}}_{2D}$, a parameter combination of m=3 and c=3 achieved an average classification accuracy greater than 85% for four out of the five classifiers, while a combination of m=3 and c=6 achieved an average accuracy greater than 85% for all the five classifiers. Similarly, for images of size 100 × 100 px, parameter combinations of m=3 and c=3,5,or6 achieved an average accuracy greater than 85% for three out of the five classifiers. We should note that the 85% classification accuracy was chosen as a threshold for the choice of parameter combinations across different time scales, classifiers, and datasets for the purpose of providing a general application to datasets and classifiers that was not explored in our experiments. Additionally, we believe there exists many techniques in which we could increase our classification accuracy, such as by bagging which can improve the stability and accuracy of classification algorithms [39]. These techniques are not explored in our work as we are only investigating the influence that key parameters exhibit on the classification performance of features extracted by the ${\mathrm{MFuzzyEn}}_{2D}$ and ${\mathrm{MDispEn}}_{2D}$ algorithms.
3. *Multiscale Analysis*: Table 2 shows the values of τ, which resulted in the highest average classification accuracy for each classifier and image size. Results show that for all five classifiers, τ>1 results in the highest average classification accuracy, indicating that complexity analysis provides a stronger textural description than irregularity analysis for the images found in this biomedical dataset.

#### 3.1.2. Computation Time

As can be seen from the algorithm mentioned in Section 2.2.3, the computation time for the ${\mathrm{MFuzzyEn}}_{2D}$ algorithm is invariant to changes in parameter values. In contrast, the computation time of ${\mathrm{MDispEn}}_{2D}$ increases as the values of *m* and *c* increase. The results show that ${\mathrm{MFuzzyEn}}_{2D}$ was computationally faster than ${\mathrm{MDispEn}}_{2D}$, achieving an average computation time of 0.29 and 4.12 seconds/per image for image sizes of 50 × 50 px and 100 × 100 px, respectively. In comparison, ${\mathrm{MDispEn}}_{2D}$ produced an average computation time of 15.18 and 26.10 seconds/per image for image sizes of 50 × 50 px and 100 × 100 px, respectively. We should note that ${\mathrm{MDispEn}}_{2D}$ was computationally faster for lower values of *m* and *c*; however, its classification accuracy remained lower than that of ${\mathrm{MFuzzyEn}}_{2D}$. All experiments were conducted on a Desktop PC with an Intel(R) Core(TM) i7-9700K CPU @ 3.60GHz, 3600 Mhz, eight core(s), eight logical processor(s) and 32 GB DIMM RAM.

### 3.2. KTH-TIPS

The KTH-TIPS dataset is comprised of 10 unique classes (textures). A binary classification is performed on a texture vs. texture basis such that each texture is classified against disparate textures. Therefore, the classification accuracy presented in the following section displays the averaged results across nine tests, as there are nine distinct classes in relation to the class under examination. All tests were conducted on images of size 100 × 100 px for this dataset.

#### Classification Accuracy

Similarly to the experiments involving the Epistroma dataset, the multiscale entropy algorithms were compared based on their overall classification performance. The two classification metrics discussed are average accuracy, which describes the average of all classification results across all texture vs. texture tests for all the classifiers, and average max accuracy, which specifies the average of the highest classification accuracy across all the texture vs. texture tests achieved by each classifier. Secondly, the optimal choice of each parameter was assessed by recording which parameter value resulted in the highest classification accuracy for each classifier across all the texture combinations. Thirdly, the multiscale entropy algorithms were compared on a texture vs. texture basis, whereby the highest average classification accuracy for each unique texture combination was identified in addition to the multiscale entropy approach used to achieve said accuracy. Finally, multiscale analysis was conducted to determine whether complexity or irregularity provides a better textural representation of our images. This was investigated in the same manner as the one described in the Epistroma tests.

**Figure 3:**
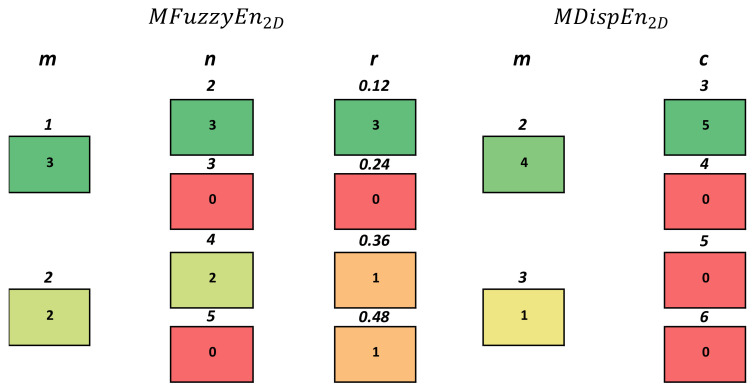
Color map displaying the performance of different parameter values as a representation of classifier performance for the KTH-TIPS dataset. The number located inside each box represents the number of classifiers (over five classifiers) that produced the highest average classification accuracy based on the respective parameter value (number above each box). Darker green boxes show the optimal parameter choice, while red boxes show the weakest choice.

**Figure 4:**
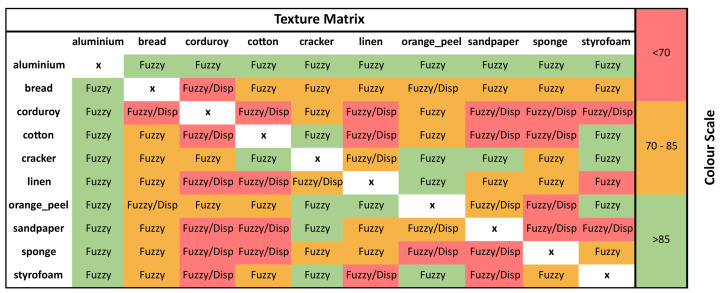
Texture matrix representing the performance of both ${\mathrm{MFuzzyEn}}_{2D}$ and ${\mathrm{MDispEn}}_{2D}$ algorithms with the KTH-TIPS dataset. The entropy technique, which achieved a higher average classification accuracy, is displayed within the matrix, while the color scale represents the average classification accuracy achieved.

1. *Classification Performance*: As can be seen in Table 3, across all the possible parameter, texture, and multiscale combinations, ${\mathrm{MFuzzyEn}}_{2D}$ outperformed ${\mathrm{MDispEn}}_{2D}$ in both average classification and max average classification accuracy. Additionally, the KNN classifier achieved the highest average and maximum average classification accuracy for both entropy techniques.
2. *Parameter Optimization*: Figure 3 shows a color map that displays the performance of different parameters with respect to each classifier for the ${\mathrm{MFuzzyEn}}_{2D}$ and ${\mathrm{MDispEn}}_{2D}$ algorithms. These results indicate that a majority of the classifiers (three out of five) achieve optimal classification performance for a parameter combination of m=1, n=2, and r=0.12. Similarly, Figure 4 indicates that lower values for the parameters *m* and *c* produced a higher classification accuracy for textures extracted by the ${\mathrm{MDispEn}}_{2D}$ algorithm. Additionally, for ${\mathrm{MFuzzyEn}}_{2D}$, all parameter combinations resulted in an average accuracy greater than 85% for all the classifiers in the experiments containing the texture aluminium, while no parameter combination resulted in an average accuracy greater than 85% for any classifier when using ${\mathrm{MDispEn}}_{2D}$.
3. *Texture Analysis*: Figure 4 displays each texture vs. texture test alongside which entropy algorithm achieved the highest average classification accuracy, where a green cell represents an average classification accuracy greater than 85%, a yellow cell represents an accuracy between 70% and 85%, and a red cell represents an accurancy of less than 70%. Results indicate that ${\mathrm{MFuzzyEn}}_{2D}$ performed extremely well in every test involving aluminium. Moreover, across the board, ${\mathrm{MFuzzyEn}}_{2D}$ outperformed or matched the classification performance of ${\mathrm{MDispEn}}_{2D}$ on all the texture combinations. Furthermore, both entropy techniques performed poorly on a majority of the tests involving the texture corduroy.
4. *Multiscale Analysis*: Table 4 displays the average classification accuracy for the multiscale extensions ${\mathrm{MFuzzyEn}}_{2D}$ and ${\mathrm{MDispEn}}_{2D}$ across different scale factors. The results show that the texture images examined in this study contained complex structures across multiple spatial scales: for τ values>1, the average classification accuracy increased for all the classifiers.

The results from our study show that the choice of optimal parameter values for both ${\mathrm{MFuzzyEn}}_{2D}$ and ${\mathrm{MDispEn}}_{2D}$ is dependent on the image category under examination. For example, for images in the biomedical dataset (Epistroma), larger parameter values are recommended as a majority of the classifiers used in our experiments performed optimally with larger values of $m,\;n,\;r\;\left({\mathrm{MFuzzyEn}}_{2D}\right)\;\mathrm{and}\;m,\;c\;\left({\mathrm{MDispEn}}_{2D}\right)$. In contrast to this, for images in the generic texture dataset (KTH-TIPS), the results advocate for lower values of parameters for both multiscale entropy algorithms.

Moreover, multiscale analysis is recommended as it demonstrated better classification results with both entropy measures. This is in agreement with the literature and considering the fact that complexity analysis is more informative and inclusive for the inherent data at several scale factors.

Furthermore, although ${\mathrm{MDispEn}}_{2D}$ is more computationally efficient at lower values of mandc, its computation time increases exponentially for larger values. Moreover, for all parameter values, the computation time for the ${\mathrm{MFuzzyEn}}_{2D}$ algorithm remained consistent, in addition to superior classification performance across both datasets. ${\mathrm{MFuzzyEn}}_{2D}$ emerges as the optimal textural feature extraction algorithm for the classification of images found in the Epistroma and KTH-TIPS datasets.

### 3.3. Main Findings and Significance of the Work

From the results mentioned above, the main findings of our study are as follows:

- Textural features extracted by ${\mathrm{MFuzzyEn}}_{2D}$ resulted in better classification performance than those extracted by the ${\mathrm{MDispEn}}_{2D}$ as a majority.
- In ${\mathrm{MDispEn}}_{2D}$, for images of size 50 × 50 px from the Epistroma dataset, parameter combinations of (1) m=3 and c=3, and (2) m=3 and c=6 achieved an average classification accuracy greater than 85% for four out of the five classifiers and for all five classifiers, respectively. For images of size 100 × 100 px, parameter combinations of m=3 and c=3,5,or6 achieved an average accuracy greater than 85% for three out of the five classifiers. Additionally, for images in the KTH-TIPS dataset, no parameter combination resulted in an average accuracy greater than 85%.
- In ${\mathrm{MFuzzyEn}}_{2D}$, for images in the Epistroma dataset, parameter combinations of (1) m=1, n=4, r=0.24,0.36,0.48, m=1, n=5, r=0.12,0.24, and 0.36, and (2) m=2, n=5, r=0.36, and 0.48 resulted in an average accuracy greater than 85% for both image sizes. Additionally, for images in the KTH-TIPS dataset, all parameter combinations resulted in an average accuracy greater than 85% for experiments containing the texture aluminium.
- The computation time of ${\mathrm{MFuzzyEn}}_{2D}$ was invariant to changes in parameter values. Contrarily, larger values of *m* and *c* increased the computation time of ${\mathrm{MDispEn}}_{2D}$ exponentially. Furthermore, ${\mathrm{MDispEn}}_{2D}$ was computationally faster than ${\mathrm{MFuzzyEn}}_{2D}$ for lower values of *m* and *c*. However, in most cases, this lowered the classification performance.
- The multiscale version of entropy measures led to the creation of a vector of entropy values. Our results reveal that, when the vector of entropy values is applied to the classifier, the subsequent results show improved classification accuracy. This shows that the texture of coarse-grained versions of images provides information for classification purposes.
- In most cases, the choice of classifier did not have a significant impact on the classification of the extracted features by both entropy algorithms.

Other authors used the Epistroma dataset and KTH-TIPS dataset to compare different texture algorithm performances. Thus, Bianconi et al. processed the color images (we processed their grayscale version) and used local binary pattern (LBP) variants for texture feature extraction, alongside CNN-based features [40]. Moreover, Bello-Cerezo et al. used the same datasets with extensions of LBP [41]. Kather et al. used the Epistroma dataset with pre-trained deep networks and LBP variants [42]. The results reported by all these studies are in the same range as the ones presented herein. Our study therefore shows that by using specific parameters, namely ${\mathrm{MFuzzyEn}}_{2D}$ and ${\mathrm{MDispEn}}_{2D}$, one can approach state-of-the-art in terms of image classification for the two datasets processed.

## 4. Conclusions

In this paper, we provided a comparison of various parameter selections for both FuzzyEn${}_{2D}$ and DispEn${}_{2D}$ in terms of classification accuracy and computation time. Additionally, this comparison was extended to the multiscale version of each algorithm (MFuzzyEn${}_{2D}$ and MDispEn${}_{2D}$). For this purpose, two publicly available datasets were processed: the Epistroma dataset and the KTH-TIPS dataset. From our review of the literature, we understand this work to be the first experimental study on the influence of parameter selection for both FuzzyE${}_{2D}$ and DispEn${}_{2D}$ using a variety of machine learning classifiers.

Our study shows that by using specific parameters, namely ${\mathrm{MFuzzyEn}}_{2D}$ and ${\mathrm{MDispEn}}_{2D}$, one can approach state-of-the-art in terms of image classification for multiple image types. However, textural features extracted by ${\mathrm{MFuzzyEn}}_{2D}$ resulted in a better classification performance than those extracted by the ${\mathrm{MDispEn}}_{2D}$ as a majority. Furthermore, the computation time of MFuzzyEn${}_{2D}$ was not modified with the changes in parameters values, unlike that of MDispEn${}_{2D}$. Finally, the use of a multiscale approach leads to improvements in the classification results. These findings provide a guide for researchers in using MFuzzyEn${}_{2D}$ and MDispEn${}_{2D}$.

Based on the work proposed herein, other bidimensional entropy measures could be also investigated as, to the best of our knowledge, there are no complete studies examining the role of parameter selection and classification accuracy for other 2D entropy measures. In addition, the computational cost of our study could be compared to that of other classification methods, as the entropy measure applications do not require processing large datasets. Furthermore, several possible directions could be also investigated using entropy measures and machine learning techniques with possible applications in the medical field, among others.

## Figures and Tables

**Table 1:** Classification accuracy comparison between ${\mathrm{MFuzzyEn}}_{2D}$ and ${\mathrm{MDispEn}}_{2D}$ for different image sizes of the Epistroma dataset.

Entropy Algorithm	Image Size	Average Accuracy	Average Max Accuracy
${\mathrm{MFuzzyEn}}_{2D}$	50 × 50	93.40	**98.84**
	100 × 100	94.15	**100**
${\mathrm{MDispEn}}_{2D}$	50 × 50	**93.66**	97.09
	100 × 100	**94.77**	96.80

**Table 2:** Comparison between values of τ that resulted in the highest average classification accuracy for each classifier with the Epistroma dataset. τ=n indicates that gathering entropy values from scale 1 to *n* leads to the highest average classification accuracy.

	50 × 50		100 × 100	
Classifier	${\mathrm{MFuzzyEn}}_{2D}$	${\mathrm{MDispEn}}_{2D}$	${\mathrm{MFuzzyEn}}_{2D}$	${\mathrm{MDispEn}}_{2D}$
Decision tree	τ=5	τ=10	τ=6	τ=7
Naive Bayes	τ=10	τ=2	τ=10	τ=5
SVM	τ=5	τ=6	τ=9	τ=9
MLP	τ=7	τ=10	τ=10	τ=10
KNN	τ=4	τ=9	τ=7	τ=4

**Table 3:** Classification accuracy comparison between ${\mathrm{MFuzzyEn}}_{2D}$ and ${\mathrm{MDispEn}}_{2D}$ for the KTH-TIPS dataset.

	Average Accuracy		Max Average Accuracy	
Classifier	${\mathrm{MFuzzyEn}}_{2D}$	${\mathrm{MDispEn}}_{2D}$	${\mathrm{MFuzzyEn}}_{2D}$	${\mathrm{MDispEn}}_{2D}$
Decision tree	79.52	67.31	100	98.22
Naive Bayes	74.99	56.26	100	95.09
SVM	80.55	66.17	100	95.69
MLP	73.78	55.40	100	97.32
KNN	83.18	71.46	100	100

**Table 4:** Comparison between values of τ that resulted in the highest average classification accuracy for each classifier with the KTH-TIPS dataset. τ=n indicates that gathering entropy values from scale 1 to *n* leads to the highest average classification accuracy.

Classifier	${\mathrm{MFuzzyEn}}_{2D}$	${\mathrm{MDispEn}}_{2D}$
Decision Tree	τ=5	τ=4
Naive Bayes	τ=5	τ=3
SVM	τ=4	τ=5
MLP	τ=5	τ=10
KNN	τ=5	τ=10
